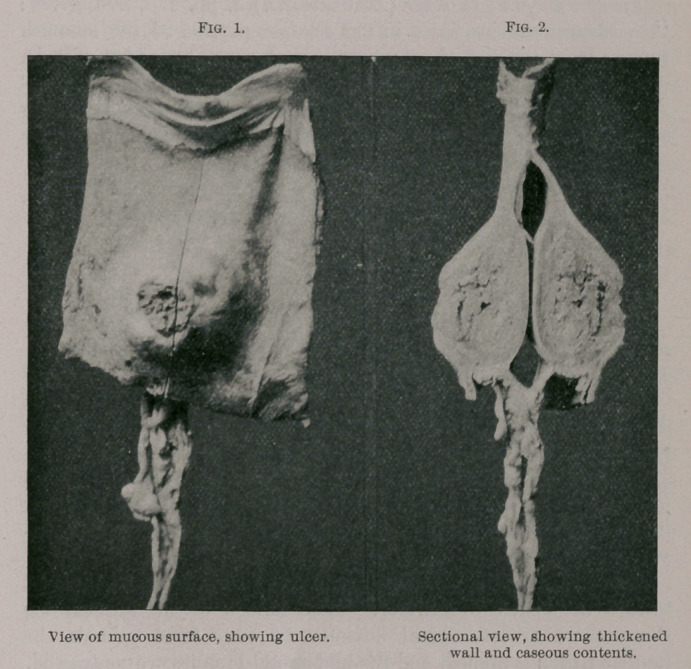# A Case of Spiroptera Megastoma (Rud)

**Published:** 1898-01

**Authors:** S. Sisson

**Affiliations:** Ontario Veterinary College, Toronto, Canada


### REPORTS OF CASES.

### A CASE OF SPIROPTERA MEGASTOMA (RUD).

By S. Sisson, V.S.,
ONTARIO VETERINARY COLLEGE, TORONTO, CANADA.

The subject of this note was a female donkey which had been condemned on account of a pelvic fracture involving the acetabulum. In demonstrating the case to the post-mortem class, a tumor was noticed projecting from the anterior face of the stomach.

Fig. 1. Fig. 2.

View of mucous surface, showing ulcer. Sectional view, showing thickened wall and caseous contents.

The omentum was adherent to it over a small area. On opening and cleansing the stomach the apparent tumor was found to be an intramural abscess, which projected somewhat further into the stomach than it did externally. The mucous membrane presented an ulcer on the summit of the abscess about 2.5 cm. (1 in.) in diameter, through which appeared a few *spiroptera*, partially embedded in the caseous pus which filled the abscess-cavity.

The meaurements of the abscess were as follows: diameter, 7.5 cm. (3 in.); circumference, measured internally, 21.5 cm; externally, 23.5 cm.; thickness from within to without, 4 cm.

Although the writer has had an opportunity to examine several hundred stomachs, many of which presented spiroptera abscess, nothing of this magnitude has yet been met with. The history of the case for the two years previous was obtained without any symptoms of digestive derangement being elicited, and the general condition at death was excellent.

*Friedberger and Fröhner* (Zuill's trans.), i. pp. 275, 276, gives: "S. Megastoma produces in the *cardiac* portion of the stomach tumors from the size of a bean to that of a nut, provided with an opening and able to cause gastritis, colic, etc." So far as my experience goes they always seem to affect the *pyloric* portion of the stomach (as in this case) and do not seem to cause gastritis or any appreciable clinical signs of their presence.

BIBLIOGRAPHY.

Neumann: Treatise on Parasites. (Fleming's trans.), pp. 346, 347. Full account.
Cobbold: Parasites, London, 1879. Pp. 381, 383.

## Figures and Tables

**Fig. 1. Fig. 2. f1:**